# Comprehensive Characterization of *Tuber maculatum,* New in Uruguay: Morphological, Molecular, and Aromatic Analyses

**DOI:** 10.3390/jof10060421

**Published:** 2024-06-14

**Authors:** Francisco Kuhar, Eva Tejedor-Calvo, Alejandro Sequeira, David Pelissero, Mariana Cosse, Domizia Donnini, Eduardo Nouhra

**Affiliations:** 1 Innomy Biotech S.L. Astondo Bidea, Edificio 612, 48160 Derio, Spain; 2 Instituto Multidisciplinario de Biología Vegetal (IMBIV-CONICET), Facultad de Ciencias Exactas, Físicas y Naturales (F.C.E.F. y N.), Universidad Nacional de Córdoba, Córdoba 5000, Argentina; davidpelissero@mi.unc.edu.ar (D.P.); enouhra@gmail.com (E.N.); 3 Department of Plant Science, Agrifood Research and Technology Centre of Aragon (CITA), Avda. Montañana, 50059 Zaragoza, Spain; etejedor@unizar.es; 4 Laboratory for Flavour Analysis and Analytical Chemistry, Faculty of Sciences, University of Zaragoza, 50009 Zaragoza, Spain; 5 Departamento de Biodiversidad y Genética, Instituto de Investigaciones Biológicas Clemente Estable (IIBCE), Ministerio de Educación y Cultura (MEC), Av. Italia 3318, Montevideo 11600, Uruguay; graficatrocadero@gmail.com (A.S.); marianacosse@gmail.com (M.C.); 6 Department of Agricultural, Food and Environmental Science, University of Perugia, 06121 Perugia, Italy; domizia.donnini@unipg.it

**Keywords:** truffles, volatile compounds, phylogeny, hypogeous

## Abstract

Although only a few species of *Tuber* account for the major truffle sales volume, many species that are not considered delicacies are finding their way to the market, especially in regions where the traditionally appreciated ones do not occur. This is the case for whitish truffles. Specimens of whitish truffles were collected in pecan (*Carya illinoinensis*) orchards in Uruguay in October 2021. Morphological and molecular methods were used to characterize and assess their identity as *Tuber maculatum* Vittad. An SPME extraction of volatile compounds and GC–MS analyses were performed to characterize the aromatic profile of these specimens and evaluate their potential applications. Among the 60 VOCs detected, 3-octenone (mushroom odor), 3-octanol (moss, nut, mushroom odor), and 2H-pyran-2-one (no odor), followed by octen-1-ol-acetate (no odor) and 2-undecanone (orange, fresh, green odor) were the major compounds in *T. maculatum* fruiting bodies. The attributes of exotic edible mushrooms of commercial value in the region are highlighted. In particular, this work emphasizes the characteristics of truffles as a byproduct of pecan cultivation.

## 1. Introduction

Truffles are among the most appreciated edible fungi worldwide [1]. Their aroma, a putative adaptation to attract mammals that might act as spore dispersal agents, might be involved in complex biotic interactions and also captivates the admiration of food connoisseurs [2]. True truffles belong to the genus *Tuber*, and their diversity in Europe, North America, and Asia is well studied. However, only representatives of the puberulum lineage have naturally reached the South American continent [3,4], apparently associated with trees migrating from North America during the GABI (Great American Biotic Interchange).

A long history of tree species introduction in South America resulted in the anthropogenic migration of many fungal species. Basidiomycetes in the genera *Suillus* and *Rhizopogon* are the best-known examples, and they have even been proven to facilitate the propagation of invasive coniferous tree species [5,6]. However, representatives of the genus *Tuber* (native or introduced) are still considered rare findings in this part of the world. Romero and Blumenfeld [7], and Lorenzo and Calvelo [8], reported few species for Argentina. Intentionally introduced species have been exploited in Chile and Uruguay since 2009 and 2014, respectively [9], while at least one truffle farm was established in Argentina in 2011 [10]. Also, species accidentally implanted with their ectomycorrhizal hosts have been studied and exploited [11,12].

According to Lancellotti et al. [13], the group known as “whitish truffles” due to their light colors has representative species worldwide. These species belong to the “puberulum group” (op. cit.) and the “maculatum clade” [14] and are not considered delicacies; although, in recent years, this appreciation has been changing. One interesting example is the case of the Bianchetto Truffles (*Tuber borchii* Vittad.), whose production in areas outside their natural range is growing annually [13]. Within the maculatum clade, another interesting case exists: *Tuber floridanum* A.C. Grupe, Sulzbacher & M.E. Sm., which occurs in Brazil and is associated with pecan nut orchards. As per the local media, this species is receiving increased attention from chefs and producers, and is being sold in the market due to its interesting smell [15], reaching 6000 R$ per kg (approx. 1200 U$D) [16] (verified by Marcelo Sulzbacher, pers.com.).

European traditions and preferences frequently define the culinary value of mushroom species in the Western world. Interestingly, in countries where traditionally appreciated mushrooms do not occur, species that can be of minor or no importance in Europe usually become interesting alternatives, even among chefs. This is the case of *Suillus luteus* in Southern South America, which ranks second in market volume after the champignon (*Agaricus* spp.). However, the best example is the case of *Gymnopilus* species in Uruguay, which produce abundant fructifications on *Eucalyptus* stands. While these species are considered non-edible or even toxic in many other regions, they have found great acceptance in Uruguay. They are sold in marinade or pickle preparations after repeated blanching of the mature basidiomes to eliminate the bitterness, constituting a continuously growing commercial activity [17,18,19].

Recent findings of whitish truffles in Uruguay were received with surprise by the media and the population (see, e.g., Lagos [20]). The identification and characterization of their aromatic profiles are the first steps to evaluating the design of special seasonings or aromas for this country’s culinary sector, which always looks for new alternatives to enrich the diversity of its menu.

Pecan nut (*Carya illinoinensis*) cultivation in South America is a well-established agricultural production, especially in the Southernmost countries. Only in Uruguay, 1000 ha are dedicated to this activity, while Argentina reaches 8000, Peru 2950, and Brazil 12,000 ha [21]. The feasibility of additional activities that can contribute to higher profitability of this production would be interesting for the agriculture of these countries.

As Halász et al. [22] pointed out that studying whitish truffles in the groups mentioned above presents a particular challenge due to the unstable species delimitation and morphological similarity. These authors suggest that conflicting descriptions and focusing on misleading characters might explain why these species are often confused.

This paper aims to combine the molecular and morphological approaches to assess the identity of these recent findings in Uruguay and to provide information on the volatile compounds of the collected specimens to contribute to their potential uses in food preparations.

## 2. Materials and Methods

### 2.1. Material Collection

The specimens were collected in October 2021 in the departments of Colonia and Maldonado, Uruguay (date and coordinates indicated in the taxonomical section). The aroma was recorded, and in-situ photographs were taken before they were preserved at 10C in paper envelopes. Due to the long delivery times, they arrived at the laboratory in Córdoba, Argentina, slightly dried, which probably affected the volatile composition.

### 2.2. Morphoanatomical Characterization

The fruiting bodies were completely dried at 45 °C for preservation at the fungarium. Analysis of truffle micromorphological features was performed under a phase contrast microscope. Ascospores were separated by scraping the gleba with a razor blade and mounting the fungal tissue on a slide with 5% KOH. In total, 20 spores from numerous asci were measured and imaged at 400× magnification against the long and short axes, excluding ornamentation. Measurements of the spores were then calculated, as these metrics are informative in separating species of whitish truffles.

### 2.3. DNA Extraction and Sequencing

DNA extraction was performed according to Fazekas et al. [23]. Approximately 0.02 g of powdered dried material was transferred to Eppendorf tubes containing 200 µL of CTAB lysis buffer, 40 µL of DTT (dithiothreitol), and 10 µL of Proteinase K, mixed, and incubated for 1 h at 65 °C. Each lysate was transferred into an EconoSpin All-In-One Silica Maxi Spin Column (catalog no. 1920-250; Epoch Life Sciences, Missouri City, TX, USA) and the instructions of the provider were followed, washing with ethanol and suspending the DNA in Milli-Q water. The quality and quantity of the DNA were evaluated via a NanoDropTM ND-1000 UV–vis Spectrophotometer (Nano-Drop Technologies, Inc., Wilmington, DE, USA). A 622 bp fragment of the ITS region was amplified with the ITS1 (5′-3′ TCCGTAGGTGAACCTGCGG) and ITS4 (5′-3′ TCCTCCGCTTATTGATATGC) [24]. A PCR reaction of a 15 μL final volume was performed under the following conditions: 1× Invitrogen buffer, 1.5 mM MgCl_2_, 0.2 mg/mL BSA, 0.03 dNTPs, 0.05 U of Taq DNA polymerase, 0.1 μM of each primer, and 20–100 ng/μL of DNA. The amplification protocol consisted of an initial denaturation step at 94 °C for 1 min, followed by 35 cycles of denaturation at 94 °C for 35 s, annealing at 55 °C for 55 s, and extension at 72 °C for 45 s. The final step was the extension at 72 °C for 10 min [25]. Negative controls were used in each PCR reaction. PCR products were evaluated using 1% agarose gel electrophoresis and sent to Macrogen (Korea) The PCR products were purified and sequenced at Macrogen Inc. (Seoul, Republic of Korea) using an ABI 3730XL Sequence Analyzer for Sanger sequencing by capillary electrophoresis. The newly generated sequences were uploaded to GenBank. Reference vouchers are deposited in the CORD Herbarium (Museo Botánico Córdoba, Argentina) and the Funga Lab Collection (Instituto Clemente Estable, Montevideo, Uruguay).

### 2.4. Phylogenetic Analyses

Internal Transcribed Spacer sequences of the most similar identified vouchers were queried in GenBank NCBI database (https://www.ncbi.nlm.nih.gov (accessed on 15 January 2023)) using the MEGABLAST option (see Table 1). A sequence corresponding to *T. excavatum* was used to root the topology following the method of Halász et al. [22] The sequences were aligned using the L-INS-i strategy implemented in MAFFT 7.0 [26] and analyzed without further editions. The nucleotide substitution model TN93 was selected using jModelTest 2.1 [27], under the Akaike information criterion (AIC). Maximum likelihood (ML) analyses were performed in PHYML [28]. Bootstrap support values for the highest likelihood tree were calculated with 1000 repetitions through 10 random addition sequences, and the topologies were produced using TBR swapping. Bayesian MCMC analyses were conducted with MrBayes [29] with 8,000,000 generations initiating with a random tree and running four simultaneous chains. The first 150,000 generations were discarded as burn-in. TRACER1 (http://beast.community/tracer (accessed on 10 February 2023)) was used to verify that stationarity was achieved after the first 150,000 generations.

### 2.5. Volatile Organic Compounds Extraction by SPME

The methodological approach was based on that of Tejedor-Calvo et al. [30]. A solid-phase microextraction (SPME) was used to obtain the volatile aromatic compounds (VOCs). A fused silica fiber coated with a 50/30 mm layer of divinylbenzene/carboxen/polydimethylsiloxane from Supelco (Barcelona, Spain) was chosen for the analysis. The samples (2 g of dry truffle tissue) were placed in a 20 mL glass vial closed with a septum. After that, the vial was conditioned at 50 °C for 10 min. the fiber was exposed to the headspace of the vial for 20 min. Analyses were carried out in duplicate.

### 2.6. GC–MS Analysis

The VOC profile of the samples was analyzed using a gas chromatograph Perkin Elmer Clarus 600 Series coupled with a Perkin Elmer Clarus 600 mass spectrometer detector (Chatsworth, CA, USA). This SPME–GC–MS instrument had a capillary column HP-5MS of 30 m, 0.32 mm i.d., 0.25 μm film thickness, and a 1 mL/min flow with helium as a carrier gas. The samples were injected in the splitless mode. The oven temperature was 45 °C held for 2 min, 45–200 °C at a rate of 4 °C/min, and finally, to 225 °C at 10 °C/min, and held for 5 min. The MS used the electron impact mode with an ionization potential of 70 eV and an ion source temperature of 200 °C. The interface temperature was 220 °C. The MS scanning was recorded in full scan mode (35–250 *m*/*z*). The m/z corresponds to the three ions with the highest relative abundances in the compound out of all the ions in the sample. TurboMass software (V. 6.1.0) was used to control the GC–MS system.

### 2.7. Aromatic Data Analysis

Peak identification of the VOCs was achieved by comparison of the mass spectra with mass spectral data from the NIST MS Search Program 2.0 library and by comparison of previously reported Retention Indices (RIs) with those calculated using an n-alkane series (C6–C20) under the same analytic conditions (all standards of purity higher than 95%). A semi-quantification was carried out by integrating the area of one ion characteristic of each compound and normalization by calculating the relative percentage using the OpenChrom (V. 1.5.0) software. This allowed the comparison of each eluted compound between samples.

## 3. Results

### 3.1. Phylogeny

**Figure 1:**
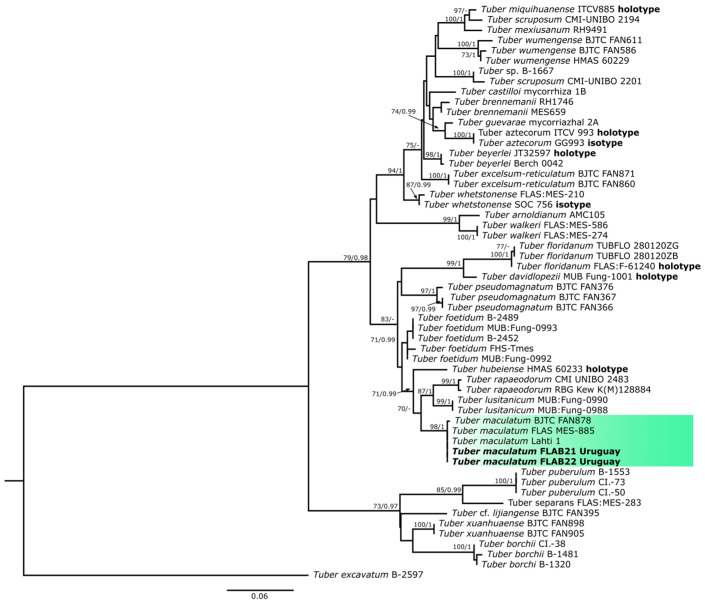
Consensus tree showing a monophyletic group (highlighted) containing *Tuber maculatum* sequences from Uruguay (ectomycorrhizal and voucher specimens among various conspecific sequences from Europe, Asia, and North America). BS and PP support values are indicated above each node. Names in bold indicate sequences from this work. *Tuber excavatum* was used as an outgroup.

Uruguayan vouchers formed a monophyletic unit with high support (98% bootstrap and 1.00 posterior probability) with other vouchered specimens of *Tuber maculatum* identified in reference works such as that of Fan et al. [31] (Figure 1). Our analyses also show a close relation with other whitish truffle species, such as *Tuber rapaeodorum* Tul. & C. Tul. and *T. lusitanicum* Ant. Rodr. & Muñoz-Mohedano. They are nested with additional species within the maculatum group, such as *T. floridanum*, *T. pseudomagnatum* L. Fan, and *T. foetidum* Vittad. Despite their morphological similarities, no close relationships with whitish truffles such as *T. borchii* and *T. puberulum* Berk. & Broome (puberulum group) are evidenced. These results confirm the identity suggested by our morphological analysis (Section 3.2).

### 3.2. Taxonomy

**Figure 2:**
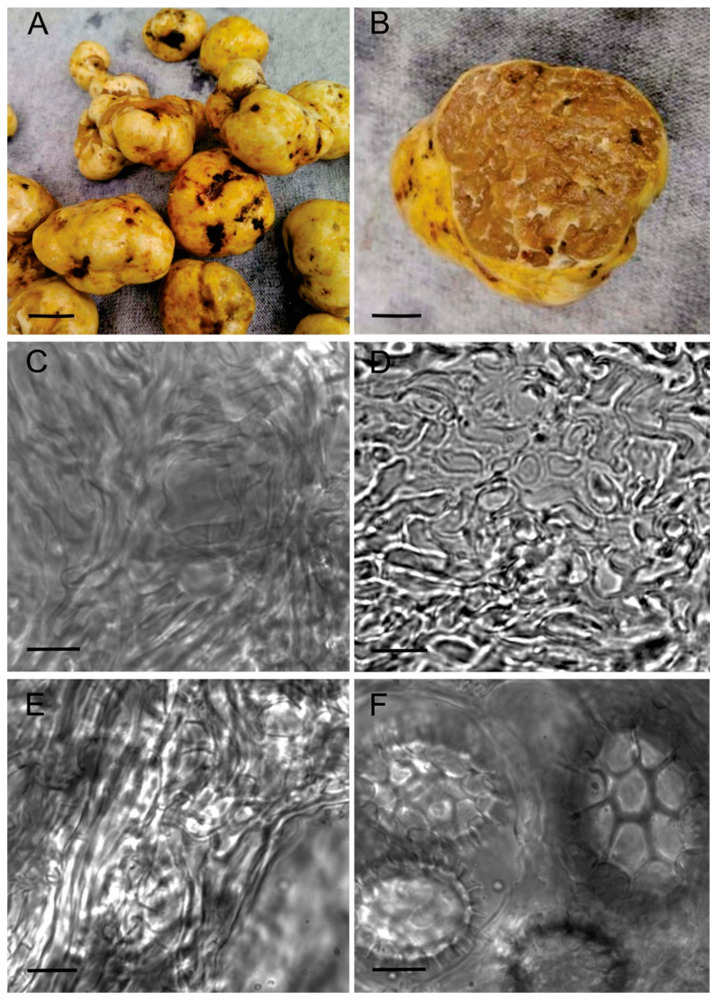
*Tuber maculatum* FLAB21. (**A**) Ascomata external view. (**B**) Ascomata in cross-section with glebal tissue. (**C**) Prosenchymatous peridial hyphae. (**D**) Thin section of the peridium. (**E**) Glebal hyphae. (**F**) Spores showing ornamentation. Bars: A = 1 cm; B = 2 cm; C, D, E, F = 10 µm.

The anatomical analysis of the specimens studied revealed that the morphological characters were consistent with those of *Tuber maculatum*. Based on our observations, we present below a brief description of the species, including the characters of the highest diagnostic value and photographs (Figure 2).

*Tuber maculatum* Vittad.

Ascomata subglobose, 0.8 to 3 cm, peridium slightly irregular, pale to yellow (mustard yellow), somewhat shiny, brownish red where bruised, also darker in the depressions, usually with adhering debris (Figure 2A). Peridium hygrophanous in patches.

Peridium 300–400 µm thick, prosenchymatous, consisting of pale yellow hyphae 2–6 µm diam. and with thin walls up to 0.5 µm thick near the surface (Figure 2C), the internal hyphae becoming hyaline (Figure 2D); emergent hyphal tips not present. Glebal hyphae generally cylindrical, confluent with the peridial hyphae, and of similar shape and size (Figure 2E). Asci 40–90 × 40–60 µm, globose to ellipsoid, some irregular, hyaline, thin-walled, 1–4 (-5), mostly four-spored, sometimes having a stem.

Spores ovoid to ellipsoid, in one-spored asci 47–60 × 38–44 µm, in two-spored asci 38–43 × 28–36 µm, in three- and four-spored asci 22–40 × 20–30 µm; light golden to light brown, ornamented with a regular reticulum 2–5 µm deep, formed by hexagonal patches; looking somewhat thorny in the optical section (Figure 2F).

Ecology and distribution: Hypogeous in loamy soil, in an orchard, Uruguay, Oct.

Specimen examined: URUGUAY. Dpto. Colonia: Conchillas (34°09′43.5″ S 58°02′05.8″ W), Col. José Ángel Latorre. Under *Carya illinoinensis*, 26 October 2021, La Torre J.A. (CORDC0014155; FLAB21) GenBank PP297667. URUGUAY. Dpto. Maldonado: (34°54′32.7″ S 54°55′54.9″ W), Col. Graciela Vega Leoncini. Under *Carya illinoinensis*, 22 October 2021, La Torre J.A. (FLAB22), GenBank PP297668.

Additional comments: *Tuber maculatum* Vitt. has been registered among other *Tuber* species in the region. It has an overall morphology similar to *T. borchii*, *T. puberulum*, and *T. rapaeodorum*. However, *T. maculatum* can be distinguished from these taxa by the presence of a prosenchymatic peridium [22,32]. In Argentina, this species was observed in the province of Córdoba [7] and reported for Patagonia [8]. In both situations, it was associated with exotic tree species. To our knowledge, this is the first record of this species in Uruguay.

### 3.3. Aromatic Profiling of T. maculatum

**Figure 3:**
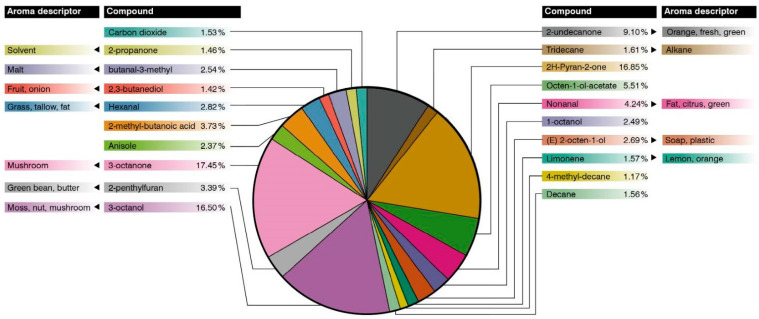
Aromatic profile of the most abundant compounds found in *Tuber maculatum* samples and their respective aroma descriptors found in the literature. Empty spaces correspond to ambiguously described aromas or compounds without available descriptions.

A total of 60 VOCs were detected in *T. maculatum* fruiting bodies, with only 20 (Figure 3) showing a concentration higher than 1% of the total compounds detected. Among them, 3-octanone, 3-octanol, and 2H-pyran-2-one were reported as the major compounds (almost 50% of the total VOCs). In the second order, octen-1-ol-acetate and 2-undecanone were detected. The *Tuber maculatum* aromatic profile differed from other truffle species. (E)-2-octen-1-ol and 3-octanol were detected as key compounds in the *T. indicum* aromatic profile [33]. However, 2H-pyran-2-one has been reported in *Tricholoma* species [34]. Some of the main VOCs detected have aromatic properties such as mushroom-like, nuts, malt, or citrus (Figure 3). Therefore, the aromatic profile of *T. maculatum* could be described as mushroom-like with some green and fruity notes. For a complete list including the minoritarian compounds, see the Supplementary Table S1.

Our collections of *T. maculatum* displayed aromatic properties more closely related to *T. melanosporum* Vittad., *T. indicum*, or *Terfezia claveryi* Chatin than other commercial truffle species such as *T. aestivum* Vittad. or *T. magnatum* Picco.

## 4. Discussion

This work reports the characterization of *Tuber maculatum* recently found in Uruguay. Molecular analyses place these materials in a monophyletic group corresponding to referenced materials of this species. The study of further collections and the revision of materials from Patagonia and other South American landscapes will help clarify the host range in non-native areas of the distribution. For example, Patagonian collections were found in orchards with *Pinus contorta* and *Betula pendula* as possible partners [8]. Also, as Halász et al. [22] pointed out, this species remarkably resembles *T. rapaeodorum* and *T. borchii*. For this reason, describing the newly found materials in the framework of molecular studies is important to discuss the delimitation and variation within this group.

Although the pungent odor of *Tuber maculatum* and other species (e.g., *T. floridanum*) means they are generally considered unsuitable for consumption as whole foods [35], the aroma of the studied collections was described as unequivocally raphanoid at the moment of the finding, which suggests a possible culinary use as a spice in diverse food preparations. In plants, glucosinolates and isothiocyanates contribute to the distinctive notes associated with raphanoid flavor/aroma perceptions [36]. Compounds in these families have been detected in small amounts in *T. magnatum* and *T. melanosporum* truffles [37]. The methods used here (headspace solid-phase microextraction, HS-SPME), introduced by Zhang and Pawliszyn [38] and successfully employed by Pelusio et al. [39], should be able to reveal these compounds in our samples. A possible explanation for not detecting these compounds would be the re-collection and initial storage of the samples, which were not carried out by trained personnel. Further findings and analyses on fresh materials are needed to confirm the characterization of the aroma profile of *T. maculatum*. It is also important to remark that mushroom flavors change after applying culinary techniques [30]. Therefore, some of these raphanoid aromas could be completely volatilized or degraded by high temperatures, and new aromas, such as those related to the Maillard reactions, can develop during the cooking process.

The series of C8 aliphatic compounds, such as 1-octen-3-one, 3-octanol, 1-octen-3-ol, (E)-2-octen-1-ol, and 3-octanone, have been reported to be the major contributors to the characteristic flavor of diverse mushrooms [33]. It is known that these C8 compounds are mainly formed by the oxidation of fatty acids (linoleic and linolenic acids) in the presence of enzymes, such as lipoxygenase and hydroperoxide lyase [40]. Among the fatty acids, oleic (40%) and linoleic (40%) acids were reported in high percentages in *T. maculatum* species from Finland [41]. According to Tejedor-Calvo et al. [42], species in the genus *Tuber* contain between 4 and 49% of linoleic acid, and 7 and 78% linolenic acid, as the main fatty acid compounds.

Some aldehydes detected, such as hexanal and nonanal, evoke green and fatty flavors. These compounds have been found in *Tuber* species and *Terfezia claveryi* [43]. The 3-methyl butanal compound (also known as isovaleraldehyde) has also been cited as one of the key aromatic compounds of black truffle (*T. melanosporum*) in Australia, enhancing the acidic aroma notes [44]. However, 2-propanone with a solvent odor has also been detected as one of the main compounds in *T. claveryi* [43].

Although simultaneous analyses under the same conditions are needed to establish differences among species, our results suggest differences in the aroma profiles of *T. maculatum* and other closely related species, such as the ones described by Strojnik et al. (*Tuber aestivum*, *Tuber magnatum*, *Tuber melanosporum*, *Tuber mesentericum*, *Tuber brumale*, *Tuber excavatum*, *Tuber rufum*, *Tuber indicum*, and *Tuber macrosporum*) [35]. However, similarity based on quantitative results of the most abundant aromatic compounds should be interpreted with caution since humans (or animals) can usually perceive one compound more intensely than others, even if its concentration is lower. Also, the differences found between our materials and the traditionally consumed truffle species would open the possibility of exploring new uses, as in the case of *T. floridanum* in Brazil [11]. Modern methodologies of volatile compound analysis need to be employed on unambiguously identified materials to determine the underlying reasons for the variation in this clade, as already shown by Strojnik et al. [35] for other species.

In our study, only two repetitions were possible due to the limited collection size. Further materials are needed to obtain more stable characterizations. Also, verifying these analyses using fresh materials would confirm the aromatic profile of South American specimens of this species.

The absence of species highly appreciated elsewhere stimulates different culinary uses for alternative species in Southern South America. This is the case of a few *Suillus* species, *Laetiporus sulphureus* or even *Gymnopilus* cf. *junonius*, which made their way to the market in the region [17,45].

Although *T. maculatum* belongs to a group of truffle species that are not widely appreciated as edible and sometimes even considered “contaminants” in traditional truffle production [46], the possibility of incorporating them into local dishes must be considered as it was for the aforementioned taxa. In the case of the closely related *T. floridanum*, which reached high prices in the Brazilian markets in the early stages of commercialization, the intervention of recognized local chefs was decisive for the outreach and the marketing of these “alternative” species of truffles (e.g., Campos [47]).

A similar situation involves the “pecan truffle” *Tuber lyonii* in North America. This species is considered an “affordable” alternative to the traditional European species. Their particular features, such as the almond flavor, are appreciated by local consumers and are already marketed in many states [48]. In addition to the culinary value of this species, local scientists have emphasized other interesting facts, like the contribution of ectomycorrhizal species to tree nutrition [49]. In this line, another point to consider in the future would be the abundance and productivity of truffles within the pecan orchards in Uruguay and elsewhere to estimate how profitable the harvest would be in the region. In addition, adjacent pecan productive areas, such as the ones close to the Parana River or the Delta Islands in Argentina, must be surveyed for truffle occurrence and productivity.

## Figures and Tables

**Table 1:** Species, voucher data, and accessions of the sequences used in the phylogenetic analyses.

Species Name (*T* = *Tuber*)	Voucher	Herbarium Code	Country	GenBank Accession Number
*T. arnoldianum*	AMC105	-	USA	OP413000
*T. aztecorum*	ITCV 993 holotype	ITCV	Mexico	NR_159044
*T. aztecorum*	GG993 isotype	FLAS	Mexico	KY271791
*T. beyerlei*	JT32597 holotype	DUKE	USA	HM485408
*T. beyerlei*	Berch 0042	-	Canada	KP972075
*T. borchii*	B-1320	-	Hungary	AJ557540
*T. borchii*	CI.-38	-	Hungary	AJ557541
*T. borchii*	B-1481	-	Hungary	AJ557542
*T. brennemanii*	MES659	FLAS	USA	MF611783
*T. brennemanii*	RH1746	FLAS	USA	MF611791
*T.* cf. *lijiangense*	BJTC FAN395	BJTC	China	OM286854
*T. davidlopezii*	MUB Fung-1001 holotype	MUB	Spain	NR_178156
*T. excavatum*	B-2597	-	Hungary	AJ557545
*T. excelsum-reticulatum*	BJTC FAN860	BJTC	China	OM265271
*T. excelsum-reticulatum*	BJTC FAN871	BJTC	China	OM265276
*T. floridanum*	FLAS:F-61240 MES654 holotype	FLAS	USA	NR_160485
*T. floridanum*	TUBFLO 280120ZB	-	Brazil	OM212443
*T. floridanum*	TUBFLO 280120ZG	-	Brazil	OM212444
*T. foetidum*	B-2452	-	Hungary	AJ557543
*T. foetidum*	B-2489	-	Hungary	AJ557544
*T. foetidum*	FHS-Tmes (fungi hypogei of Serbia)	-	Serbia	FM205704
*T. foetidum*	MUB:Fung-0992	MUB	Spain	MT621657
*T. foetidum*	MUB:Fung-0993	MUB	Spain	MT621658
*T. guevarae*	Mycorrhiza isolation 2A	-	Mexico	JF419250
*T. hubeiense*	HMAS 60233 holotype	HMAS	China	KT067688
*T. lusitanicum*	MUB:Fung-0988	MUB	Spain	MT621653
*T. lusitanicum*	MUB:Fung-0990	MUB	Spain	MT621655
*T. maculatum*	FLAS MES-885	FLAS	USA	MT156493
*T. maculatum*	Lahti 1	-	Finland	MZ389964
*T. maculatum*	BJTC FAN878	BJTC	China	OM265280
*T. maculatum*	FLAB22	-	Uruguay	PP297667
*T. maculatum*	FLAB21	-	Uruguay	PP297668
*T. mexiusanum*	RH9491	-	USA	JF419273
*T. miquihuanense*	ITCV885 holotype	ITCV	Mexico	HM485414
*T. pseudomagnatum*	BJTC FAN366	BJTC	China	OM265238
*T. pseudomagnatum*	BJTC FAN367	BJTC	China	OM265239
*T. pseudomagnatum*	BJTC FAN376	BJTC	China	OM265241
*T. puberulum*	B-1553	-	Hungary	AJ557530
*T. puberulum*	CI.-73	-	Hungary	AJ557535
*T. puberulum*	CI.-50	-	Hungary	AJ557536
*T. rapaeodorum*	CMI UNIBO 2483	CMI-UNIBO	Armenia	DQ011849
*T. rapaeodorum*	RBG Kew K(M)128884	RBG kew		EU784429
*T. scruposum*	CMI-UNIBO 2201	CMI-UNIBO	Armenia	DQ011845
*T. scruposum*	CMI-UNIBO 2194	CMI-UNIBO	Armenia	DQ011848
*T. separans*	FLAS:MES-283	FLAS	USA	MT156443
*T. walkeri*	FLAS:MES-274	FLAS	USA	MT156441
*T. walkeri*	FLAS:MES-586	FLAS	USA	MT156452
*T. whetstonense*	SOC 756 OSC 111412 isotype	OSC	USA	AY830855
*T. whetstonense*	FLAS:MES-210	FLAS	USA	MT156439
*T. wumengense*	HMAS 60229	HMAS	China	KT067687
*T. wumengense*	BJTC FAN586	BJTC	China	OM265254
*T. wumengense*	BJTC FAN611	BJTC	China	OM265259
*T. xuanhuaense*	BJTC FAN905	BJTC	China	MK045650
*T. xuanhuaense*	BJTC FAN898	BJTC	China	MK045651
*T.castilloi*	Mycorrhiza isolation 1B	-	Mexico	JF419247
*Tuber sp.*	B-1667	-	Hungary	AJ557539

## Data Availability

The original contributions presented in the study are included in the article/Supplementary Material, further inquiries can be directed to the corresponding author.

## Supplementary Materials

The following supporting information can be downloaded at: https://www.mdpi.com/article/10.3390/jof10060421/s1, Table S1: List of detected volatile organic compounds in *Tuber maculatum* from Uruguay.
