# Supplementary material for: Comprehensive Characterization of Tuber maculatum, New in Uruguay: Morphological, Molecular, and Aromatic Analyses
Source: J Fungi (Basel). 2024 Jun 14;10(6):421. doi: 10.3390/jof10060421 (PMC11205242; doi:10.3390/jof10060421)
Supplement: Supplementary file 1 [file jof-10-00421-s001.zip › jof-2968823-supplementary.pdf]

Supplementary Table S1. List of detected volatile organic compounds in Tuber maculatum from Uruguay.

Number	Compound name	CAS number	Retention time (RT)	Index experimental	Index literature	Odor descriptor[a]	Main m/z			Sample 1	Sample 2
1	Carbon dioxide	124-38-9	1.524	588	-		44	40	44	1.01	1.75
2	Acetaldehyde	75-07-0	1.599	593	-	pungent, ethereal	44	43	42	0.08	0.00
3	Ethanol	64-17-5	1.694	600	459	sweet	45	43	47	0.72	0.77
4	2-propanone	67-64-1	1.749	604	-	caramellic, burnt	43	58	42	2.57	0.06
5	butanal-3-methyl	590-86-3	2.569	666	659	malt	41	44	39	0.01	0.01
6	butanal-2-methyl	96-17-3	2.679	674	671	cocoa, almond	57	86	41	3.32	1.25
7	1-methoxy-2-propanol	107-98-2	2.749	679	673		45	43	47	1.80	0.06
8	Pentanal	110-62-3	2.994	698	697	almond, malt, pungent	44	41	58	0.01	0.00
9	2-methyl-pentenal	123-15-9	3.769	741	-		43	58	57	0.81	0.13
10	3-methy-3-pentanol	77-74-7	3.86	746	-		54	43	73	0.35	0.03
11	2-ethyl-4-pentenal	5204-80-8	3.955	751	-		41	55	84	0.01	0.02
12	2,3-butanediol	513-85-9	4.395	775	779	fruit, onion	45	43	57	0.01	0.03
13	2-octene	111-67-1	4.67	790	810		43	41	55	0.01	2.56
14	Hexanal	66-25-1	4.84	800	800	grass, tallow, fat	44	56	41	0.62	0.10
15	2,4-dimethyl-heptane	2213-23-2	5.375	818	818		43	85	57	4.99	0.09
16	1,3-octadiene	1002-33-1	5.47	822	826		54	67	41	0.00	0.09
17	4-methyl-octane	2216-34-4	6.586	861	964		43	41	85	0.31	0.27
18	1-hexanol	111-27-3	6.711	865	870	resin, flower, green	56	43	42	0.05	0.45
19	2-methyl-butanoic acid	116-53-0	7.006	876	868		74	41	57	0.01	0.01
20	4-methyl-2-hexanone	105-42-0	7.346	888	-		43	58	41	0.03	0.18
21	Heptenal	111-71-7	7.686	900	899	fat, citrus, rancid	43	41	70	6.69	0.03
22	Butyrolactone	96-48-0	7.951	907	908		42	41	86	1.18	0.40
23	Anisole	100-66-3	8.201	914	-		108	65	78	0.67	0.79
24	Methyl hexanoate	106-70-7	8.501	923	924	fruit, fresh, sweet	74	43	87	1.51	0.00
25	4-methyl-2-heptanone	6137-06-0	8.956	936	-		43	58	59	3.45	0.82

26	Benzaldehyde	100-52-7	9.597	954	960	almond, burnt sugar	77	106	105	0.01	0.05
27	4-methyl-nonane	17301-94-9	9.822	961	961		57	43	41	0.00	0.02
28	2-methyl-nonane	871-83-0	9.917	964	966		57	71	43	0.26	0.55
29	3-octanone	106-68-3	10.657	985	984	mushroom	55	43	71	0.02	0.02
30	2-penthylfuran	3777-69-3	10.862	991	993	green bean, butter	81	82	53	0.05	0.06
31	3-octanol	589-98-0	11.022	995	993	moss, nut, mushroom	59	55	83	0.01	0.02
32	Decane	124-18-5	11.202	1001	-		43	57	41	12.45	18.98
33	Octanal	124-13-0	11.247	1002	1001	fat, soap, lemon, green	43	57	44	3.12	2.98
34	2-ethyl-2-hexenal	645-62-5	11.392	1006	1010		55	41	39	13.56	16.16
35	4-methyl-decane	2847-72-5	11.543	1010	-		43	71	57	1.13	1.69
36	2,5-dimethyl-nonane	17302-27-1	11.928	1020	-		57	43	41	0.56	0.00
37	2,6-dimethyl-nonane	17302-28-2	12.068	1024	1022		43	71	57	0.28	0.29
38	Limonene	138-86-3	12.203	1028	1026	lemon, orange	68	93	79	2.10	0.00
39	2-ethyl-1-hexanol	104-76-7	12.243	1029	1025		57	41	43	0.28	0.50
40	3-octen-2-one	1669-44-9	12.568	1038	1036	nut, crushed bug	55	43	111	0.17	0.02
41	(E) 3-octen-2-one	18402-82-9	12.538	1037	1034		55	43	111	1.64	1.20
42	Undecane	1120-21-4	13.323	1058	-	alkane	57	43	71	1.04	0.00
43	4-methyl-decane	2847-72-5	13.438	1061	1057		43	71	57	0.00	0.14
44	Dodecane	112-40-3	13.553	1065	-	alkane	43	57	71	0.23	0.01
45	(E) 2-octen-1-ol	18409-17-1	13.648	1067	1064	soap, plastic	57	41	55	0.12	0.13
46	1-octanol	111-87-5	13.758	1070	1070		41	43	55	0.00	0.21
47	2,3-dimethyldecane	17312-44-6	14.694	1096	-		43	71	57	0.11	0.09
48	Nonanal	124-19-6	14.924	1102	1102	fat, citrus, green	57	41	55	1.90	2.95
49	Octen-1-ol-acetate	77149-68-9	15.269	1120	-		43	99	54	1.36	3.13
50	2H-Pyran-2-one	2381-87-5	16.775	1153	-		82	39	54	0.01	0.02
51	2-methyl-undecane	7045-71-8	17.175	1164	1164		43	57	71	2.50	5.14
52	2-decanone	693-54-9	18.135	1191	1192		58	43	71	3.78	6.14
53	Tridecane	629-50-5	18.465	1200	-	alkane	57	43	71	15.11	15.25
54	2,6-dimethyl-undecane	17301-23-4	18.966	1215	1216		57	43	71	0.16	0.19

55	Tetradecane	629-59-4	20.056	1246	-	alkane	57	43	71	0.25	0.47
56	Benzene, m-di-tert-butyl-	1014-60-4	20.326	1254	-		175	57	41	1.18	1.72
57	2-undecanone	112-12-9	21.622	1292	1291	orange, fresh, green	43	58	71	0.26	0.37
58	4,6-dimethyl-dodecane	61141-72-8	21.737	1295	-		57	71	43	0.04	0.05
59	2,6,11-trimethyldodecane	31295-56-4	22.802	1328	-		57	71	43	0.16	0.17
60	(+)-Cuparene	16982-00-6	28.489	1508	1504		132	131	145	0.27	0.32

[a] Odor descriptor from http://flavornet.org/flavornet.html
